# Redefining attrition in multiple myeloma (MM): a Canadian Myeloma Research Group (CMRG) analysis

**DOI:** 10.1038/s41408-023-00883-x

**Published:** 2023-07-20

**Authors:** Arleigh McCurdy, Hira Mian, Richard LeBlanc, Victor H. Jimenez-Zepeda, Jiandong Su, Esther Masih-Khan, Alissa Visram, Martha Louzada, Kevin Song, Darrell White, Michael Sebag, Julie Stakiw, Anthony Reiman, Muhammad Aslam, Debra Bergstrom, Rami Kotb, Rayan Kaedbey, Engin Gul, Donna Reece, Christopher P. Venner

**Affiliations:** 1 Ottawa Hospital Research Institute, The Ottawa Hospital, Ottawa, Ontario Canada; 2 Juravinski Cancer Center, Hamilton, Ontario Canada; 3 Maisonneuve-Rosemont Hospital Research Centre, University of Montreal, Montreal, Quebec Canada; 4 Arnie Charbonneau Cancer Institute, University of Calgary, Calgary, Alberta Canada; 5 Canadian Myeloma Research Group, Vaughn, Ontario Canada; 6 Princess Margaret Cancer Centre, Toronto, Ontario Canada; 7 Department of Medicine, London Regional Cancer Center, London, Ontario Canada; 8 BC Cancer Agency, Vancouver General Hospital, Vancouver, British Columbia Canada; 9 Queen Elizabeth II Health Sciences Centre. Dalhousie University, Halifax, Nova Scotia Canada; 10 Department of Medicine, McGill University, Montreal, Quebec Canada; 11 Saskatoon Cancer Centre, University of Saskatchewan, Saskatoon, Saskatchewan Canada; 12 Department of Medicine, Saint John Regional Hospital, Saint John, NB Canada; 13 Muhammad Aslam, Department of Medical Oncology, Allan Blair Cancer Centre, Regina, Saskatchewan Canada; 14 Division of Hematology, Memorial University of Newfoundland, St John’s, Newfoundland and Labrador Canada; 15 Department of Medical Oncology & Hematology, Cancer Care Manitoba, Winnipeg, Manitoba Canada; 16 Department of Medicine, Jewish General Hospital, Montreal, Quebec Canada; 17 Department of Medicine, Cross Cancer Institute, Edmonton, Alberta Canada

**Keywords:** Myeloma, Myeloma, Cancer epidemiology

## Abstract

While most patients diagnosed with multiple myeloma (MM) receive initial therapy, reported attrition rates are high. Understanding attrition rates and characteristics of patients not receiving subsequent therapy is useful for MM stakeholders. We performed an analysis of attrition rates in a large disease-specific database of patients with newly diagnosed MM who received at least one line of therapy between Jan 1/10-Dec 31/20. Attrition was defined as failure to receive a subsequent line of therapy despite progression of MM or due to death. A total of 5548 patients were identified, 3111 autologous stem cell transplant (ASCT) patients and 2437 non-ASCT. In the ASCT cohort, the attrition rate was 7% after line 1, 12% after line 2, and 23% after line 3. In non-ASCT patients, the attrition rate was 19% after line 1, 26% after line 2, and 40% after line 3. Death was the dominant contributor to attrition across all cohorts, with a minority of patients alive with progressive disease in the absence of further therapy at each line. Multivariable analysis identified older age, shorter time to progression, and inferior response as independent risk factors for attrition. Our data show that attrition rates increase with each line of therapy and are higher in non-ASCT patients but are appreciably lower than previously reported. This study supports a revision of the previous definition of attrition, demonstrating that most patients who do not receive subsequent therapy are either continuing their current therapy and/or are in remission off-treatment rather than being irreversibly lost to attrition.

## Introduction

Multiple myeloma (MM) is a hematologic neoplasm characterized by a clonal proliferation of plasma cells in the bone marrow. Despite significant advances in treatment over the last decade, MM currently remains incurable for the majority of patients [1]. The course of the illness in MM is one of treatment, followed by a period of remission, and subsequently disease relapse requiring additional therapy. Throughout the disease course, the periods of remission become shorter as MM becomes biologically more complex and refractory to treatment [2]. The novel agents, including proteasome inhibitors (PIs), immunomodulatory agents (IMiDs), and most recently monoclonal antibodies (MAbs), have revolutionized MM treatment and resulted in unprecedented improvement in survival [3].

The PI bortezomib (BOR), IMiD lenalidomide (LEN), and first-generation MAb daratumumab (DARA) are used as treatment at diagnosis and at first relapse, in various combinations based on the efficacy shown in large randomized trials [4–7]. Treatment for second and third relapses and beyond is less standardized. This is due to the heterogeneity of the illness, challenges in defining an optimal sequencing of the available agents, and an incomplete understanding of which patients ultimately receive further therapy and which patients are lost to attrition. This data is increasingly paramount, as multiple new therapeutic options are entering the treatment landscape, including novel cellular therapies and immunotherapeutic platforms which are first-in-class with no “standard” comparator. Further, pharmacoeconomic analyses are challenging with incomplete or inaccurate estimates of the number and characteristics of patients who would be candidates for these treatments.

While previous real-world data has demonstrated that the majority of patients diagnosed with MM receive first-line therapy (64–95%), these studies also suggest that the proportion of patients who go on to receive further therapy decreases with each relapse, with only 50–61% of patients receiving second-line treatment, 17–38% third-line, 15% fourth-line therapy, and just 1% receiving fifth-line treatment [8–10]. The largest real-world retrospective study of 24,825 patients using 3 US administrative databases from 2007–2018 suggested that in ASCT-eligible patients, 21% of patients were lost to attrition prior to second-line therapy. Of those who did receive further treatment, 54% were lost prior to third-line therapy and 89% lost prior to fourth-line therapy [11]. The proportion of patients lost to attrition was even higher in the ASCT-ineligible group, with 57% of patients lost prior to second-line. Of those that proceeded to further treatment, an additional 46% (cumulative 87%) did not receive third-line therapy and 43% (cumulative 93%) were lost prior to fourth-line therapy [11]. However, studies using administrative databases likely overestimated attrition as it can be challenging in these databases to capture reasons other than death for failing to receive a subsequent treatment. The most important of these include patients treated with planned fixed-duration initial therapy who remain in remission or those undergoing continuous first-line therapy who have maintained their response.

A recent systematic review of post-protocol therapies and attrition restricted to randomized controlled trials (RCTs) also highlights that many patients do not proceed to have further treatment. This study showed that 45 of 103 RCTs (43.7%) reported on subsequent therapies, of which 27 clearly reported the patients who went on to further therapies. In RCTs of newly diagnosed patients, 5150/9351 (54.9%) went on to a second therapy. In RCTs involving relapsed patients, 2197/4501 (48.8%) of those in the control arms were treated with subsequent therapy [12]. Unfortunately, these RCTs did not report subsequent therapies line-by-line, and so further clarity on how many patients ultimately received third- or fourth-line therapy and beyond is not possible with this dataset. In addition, the proportion of PFS events that were death as opposed to progression was not reported in the RCTs, resulting in an unknown proportion of patients not receiving further lines of therapy due to still being in remission and/or on treatment.

As such, an improved understanding attrition is needed, particularly in view of highly active novel therapeutics used in heavily pretreated patients. It is unclear if attrition rates as previously reported using administrative databases accurately reflects true attrition rates in MM patients and may, in fact, be overestimating them. Using a disease-specific database that captures individual patient level data longitudinally may offer better clarity. Concern about attrition supports using the most active regimens upfront to maximize their utility, and it is possible that doing so may in fact improve attrition rates. An accurate understanding and definition of attrition in MM will allow for improved analyses of the volume and characteristics of patients who may be candidates for novel therapies in the relapsed setting. We therefore performed an analysis of attrition rates in patients with MM using the Canadian Myeloma Research Group Database (CMRG-DB).

## Methods

We conducted a retrospective observational study using the MM-specific CMRG-DB. This project was approved by the Ottawa Hospital Research Ethics Board in keeping with the CMRG-DB governance structure. Data was collected from the CMRG-DB, which is a prospectively maintained web-based centralized disease-specific database of over 8600 patients with MM at 16 Canadian institutions and includes legacy data from 2007.

Adult patients with newly diagnosed MM who received at least one line of therapy between Jan 1, 2010–Dec 31, 2020 were included in this analysis. Patients with amyloidosis or plasma cell leukemia were excluded. Patients were stratified by time of therapy initiation (2010–15, 2016–20) and category (ASCT patients, non-ASCT patients). The primary objective was to evaluate attrition at each line of therapy. Attrition was defined as failure to receive a subsequent line of therapy due to (1) death or (2) despite progression of MM in patients alive at the time of last follow-up. A line of therapy was defined as the administration of ≥1 anti-MM agent that continued until it was discontinued ≥60 days or until a new agent was administered. Post ASCT maintenance was considered part of first-line therapy. The secondary objective was to identify factors associated with attrition at each line of therapy.

### Statistical analysis

Descriptive statistics were used to report standard baseline characteristics of all MM patients in the database. Categorical variables were summarized with counts and percentages. Continuous variables were summarized with means, standard deviation, medians and/or ranges as appropriate.

Time to event analyses were used to calculate the median progression-free survival (PFS) and median follow-up time at each line of therapy. The cohort was stratified by two-time cohorts (2010–2015, 2016–2020), and two treatment cohorts (ASCT-eligible, ASCT-ineligible). The analysis was preformed according to the Kaplan–Meier/reverse Kaplan–Meier methods and the differences between median PFS and median follow-up time was assessed using the log-rank test.

The attrition rate was calculated and evaluated at each line of therapy and stratified by two-time cohorts (2010–2015, 2016–2020), and two treatment cohorts (ASCT-eligible, ASCT-ineligible). Bar charts were plotted to illustrate the patients’ evolution by cohort, ASCT and lines of treatments, with attrition rates highlighted in each graph.

Logistic regression models were constructed to evaluate the factors associated with outcome of attrition in each line of treatment stratified by eligibility of ASCT as first-line therapy and era of first-line therapy initiation (2010–2015, 2016–2020). Odds ratio (OR) and 95% confidence intervals were presented, OR > 1 with a significant p-value indicated significant contribution of that variable on the rate of attrition.

Due to the nature of real-world data collection, there were some variables with missing values. Missing values were assigned to an “unknown” category and included in the multivariable regression to preserve the statistical power of regression models.

Statistical analyses were performed using R core team 2020 (R-4.1.1), Vienna, Austria and RStudio team 2019 (RStudio-1.4.1717), Boston, MA, USA for Windows. All *p*-values were 2-sided and for the statistical analyses, *p* < 0.05 was considered to indicate a statistically significant result.

## Results

A total of 5548 patients were identified, 3111 ASCT patients 2437 non-ASCT patients. Baseline patient characteristics are presented in Table 1. The median age at first-line treatment was 60 years in the ASCT patients and 73 years in the non-ASCT patients. In the ASCT cohort, 30% of patients had ISS stage III disease and 35% had high-risk cytogenetics. In the non-ASCT patients, 44% had ISS stage III with 28% having high-risk cytogenetics.

**Table 1:** Baseline characteristics.

Characteristic	ASCT (*n* = 3111)	Non-ASCT (*n* = 2437)	Total (*n* = 5548)
*Age, median (range) at treatment initiation*			
Line 1	60 (25–77)	73 (32–99)	65 (25–99)
Line 2	62 (26–80)	75 (35–102)	68 (26–102)
Line 3	63 (34–82)	76 (36–97)	68 (34–97)
Line 4	63 (36–78)	76 (37–89)	67 (36–89)
*MM Subtype, n (%)*^a^			
IgG	1860 (64.3)	1455 (59.7)	3315 (59.8)
IgA	628 (21.7)	516 (22.8)	1144 (20.6)
IgM	6 (0.21)	19 (0.84)	25 (1.0)
FLC	399 (13.8)	276 (12.2)	675 (12.2)
*ISS Stage at diagnosis,* *n* *(%)*^a^			
I	897 (28.8)	389 (19.1)	1286 (27.3)
II	963 (31.0)	748 (36.7)	1711 (36.3)
III	812 (26.0)	902 (44.2) 1137	1714 (36.4)
Missing	439	398	837
*Cytogenetics, n (%)*^a,b^			
High-risk	587 (35.0)	321 (28.2)	908 (32.2)
Standard risk	1092 (65.0)	816 (71.8)	1908 (67.)
Missing	1432	1300	2732

^a^Based on evaluable patients.

^b^High risk cytogenetics based on the presence of any of deletion 17p, t(4;14), t(14;16).

The median follow-up in months (range) for the ASCT cohort was 44.3 (19.9–75.6) for first-line treatment, 24.9 (10.3–46.8) for second line, and 15.8 (6.4–31.1) for third line. In the non-ASCT group, median follow-up in months (range) was 29.9 (13.3–53.7) for first line, 20.8 (8.9–38.7) for second line, and 10.8 (4.4–25.3) for third line.

**Fig. 1:**
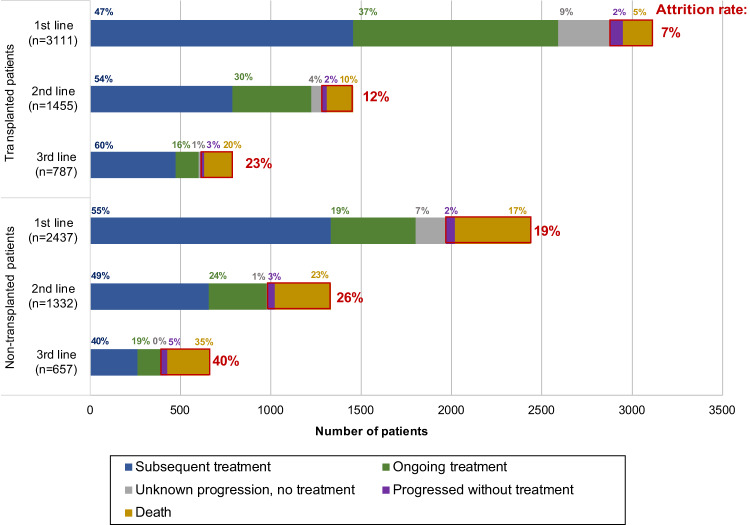
Attrition rate by ASCT Status. Disposition of transplanted and non-transplanted patients with each subsequent line of treatment. Attrition with each line of therapy is shown in red, and the percentage of attrition is the percent of patients treated at each line of therapy that either died or progressed and did not receive subsequent treatment.

The rates of attrition after each line of therapy are presented in Fig. 1. Attrition rates were higher in non-ASCT patients as compared to ASCT patients and increased in both groups with each line of treatment, with death being the main cause of attrition. After first-line treatment in the ASCT cohort, the attrition rate was 7% (5% death, 2% progression, and no further treatment). Forty-seven percent of patients went on to receive second-line treatment, 37% either remained on first-line treatment or were in remission off treatment, and 9% were lost to follow-up. After second-line treatment, the attrition rate was 12% (10% death, 2% progression, and no further treatment), with 54% of patients going on to further therapy, 30% remaining on treatment/in remission off treatment, and 4% lost to follow-up. After third-line treatment, the attrition rate was 23% (20% death, 3% progression, and no further treatment), with 60% of patients going onto further treatment, 16% remaining on treatment/in remission off treatment and 1% lost to follow-up.

In the non-ASCT patients, the attrition rate was 19% after first-line treatment (17% death, 2% progression, and no further treatment). Fifty-five percent of patients moved to second-line treatment and 19% remained on treatment/in remission off treatment, with 7% lost to follow-up. After second-line treatment, the attrition rate was 26% (23% death, 3% progression, and no further treatment), with 49% having further treatment, 24% remaining on treatment/in remission off treatment, and 1% lost to follow-up. Finally, after third-line treatment the attrition rate was 40% (35% death, 5% progression, and no further treatment), with 57% of patients having subsequent treatment, 19% remaining on treatment/in remission off treatment, and <1% lost to follow-up.

To evaluate for differences in follow-up duration and access to novel agents in recent years, we subsequently analyzed attrition rates in two time cohorts: 2010–2015 and 2016–2020. For the ASCT patients, median follow-up time in months (range) for the 2010–2015 cohort were: 71.3 (35.9–95.1) for line 1, 22.4 (9.7–41.3) for line 2 and 13.6 (5.5–26.7) for line 3, whereas in the 2016–2020 cohort the median follow-up was 26.4 (12.6–44.7) for line 1, 23.4 (10.0–46.7) for line 2, and 13.9 (5.1–28.8) for line 3. In the non-ASCT 2010–2015 cohort, median follow-up duration in months (range) was 39.2 (14.1–74.6) for line 1, 21.6 (8.9–40.4) for line 2, and 12.6 (4.7–26.4) for line 3, whereas in 2016–2020 cohort, median follow-up was 25.0 (12.6–40.8) for line 1, 23.2 (10.0–23.2) for line 2, and 14.6 (6.1–29.8) for line 3.

Trends in attrition with subsequent lines of therapy were similar to the overall cohort and results are presented in Supplementary Figure 1. Despite longer follow-up time in the more mature 2010–2015 cohort, attrition rates remained similar. In the ASCT patients, the attrition rates in the 2010–2015 cohort were 11%, 12%, and 22% for first, second, and third line, respectively, as compared to 4%, 12%, and 26% in the 2016–2020 group. In the non-ASCT patients, attrition rates were 21%, 28%, and 41% for first, second, and third line in the 2010–2015 cohort, as compared to 17%, 24%, and 39% in the 2016–2020 cohort. The main difference in the distribution of patients over the two time cohorts is that in the more mature cohort, more patients moved on to receive the next line therapy and fewer remained on their current treatment.

Multivariable analysis was performed to identify factors associated with attrition at each line of therapy by transplant status, and results are presented in Table 2. After adjusting for sex, age, PFS after first-line therapy, and depth of response, the more recent treatment cohort (2016–2020) was associated with reduced risk of attrition after first-line treatment in both transplant and non-transplant groups: ASCT odds ratio (OR) 0.45 (95% CI; 0.33–0.63) and non-ASCT OR 0.70 (95% CI; 0.56–0.87). Similarly, patients diagnosed between 2016–2020 had a lower OR of attrition after second-line therapy compared to patients diagnosed between 2010 and 2015, after adjusting for confounders (OR 0.72, 95% CI 0.55–0.94). Older age correlated with an increased risk of attrition at each line of therapy in the ASCT cohort, whereas older age was associated with increased attrition only after lines 1 and 2 in the non-ASCT group. A shorter time to progression was also associated with increased risk of attrition while there was a trend for a decreased risk of attrition in patients experiencing deeper responses.

**Table 2:** Multivariable Model showing factors associated with attrition at each line of therapy by ASCT status.

LOT	Predictors	ASCT (*N* = 3111)	Non-ASCT (*N* = 2437)
		OR (95% CI)	OR (95% CI)
1	Sex:		
	Female Male	Ref **1.33 (1.00–1.78)**	Ref 1.20 (0.96–1.49)
	Age^a^:		
	<65 65–74	Ref **1.56 (1.16–2.09)**	Ref 0.95 (0.68–1.31)
	75–84	0 (0–Inf)	**1.54 (1.11–2.13)**
	>=85	0 (0–Inf)	**3.57 (2.38–5.35)**
	Cohort:		
	2010–2015 2016–2020	Ref **0.45 (0.33–0.63)**	Ref **0.70 (0.56–0.87)**
	Progression time (m):		
	≥24 12–24	Ref **1.59 (1.10–2.31)**	Ref **0.54 (0.38–0.79)**
	6–12	**2.81 (1.71–4.63)**	1.32 (0.94–1.86)
	<6	1.10 (0.51–2.36)	**3.45 (2.54–4.70)**
	Response:		
	<PR ≥PR	Ref 0.61 (0.35–1.08)	Ref **0.75 (0.56–1.00)**
2	Sex:		
	Female Male	Ref **1.44 (1.02–2.02)**	Ref **1.40 (1.08–1.81)**
	Age^a^:		
	<65 65–74	Ref **1.44 (1.01–2.07)**	Ref 1.01 (0.68–1.50)
	75–84	0 (0–Inf)	1.20 (0.80–1.79)
	>=85	0 (0–Inf)	**2.97 (1.72–5.15)**
	Cohort:		
	2010–2015 2016–2020	Ref 0.97 (0.68–1.40)	Ref **0.72 (0.55–0.94)**
	Progression time (m):		
	≥24 12–24	Ref 1.52 (0.83–2.76)	Ref 1.20 (0.81–1.77)
	6–12	**2.00 (1.17–3.43)**	1.34 (0.89–2.01)
	<6	**4.63 (3.07–7.00)**	**2.48 (1.78–3.46)**
	Response:		
	<PR ≥PR	Ref 1.40 (0.91–2.17)	Ref 0.76 (0.55–1.04)
	Gender: Female	Ref	Ref
	Male	1.11 (0.78–1.58)	1.13 (0.81–1.58)
3	Age^a^:		
	<65 65–74	Ref **1.78 (1.20–2.62)**	Ref 0.98 (0.60–1.62)
	75–84	0 (0–Inf)	1.04 (0.62–1.73)
	>=85	0 (0–Inf)	2.32 (0.92–6.01)
	Cohort:		
	2010–2015 2016–2020	Ref 1.13 (0.74–1.72)	Ref 0.92 (0.64–1.31)
	Progression time (m):		
	≥24 12–24	Ref 1.08 (0.51–2.27)	Ref **3.12 (1.74–5.59)**
	6–12	1.62 (0.92–2.84)	**2.44 (1.41–4.23)**
	<6	**2.80 (1.73–4.51)**	**3.94 (2.48–6.27)**
	Response:		
	<PR ≥PR	Ref 0.90 (0.90–1.37)	Ref **0.57 (0.38–0.86)**

Values bolded *p* < 0.05.

^a^Age at therapy initiation.

We also performed multivariable analysis stratified by the two time periods of diagnosis (2010–2015 and 2016–2020) to evaluate for differences in follow-up duration and better access to novel agents in recent years, which showed similar results to the overall cohort and is presented in Supplementary Table 1.

## Discussion

In this large observational study, we show that attrition rates increase with each line of myeloma therapy and are higher in ASCT-ineligible patients but, notably, are appreciably lower than what has been previously reported.

When comparing our results to the large US administrative database study [11], rates of attrition in our study are considerably lower at each line of treatment for both ASCT patients (first-line 7% vs. 21%, second-line 12% vs. 31%, third-line 23% vs. 37%) and non-ASCT patients (first-line 19% vs. 57%, second-line 26% vs. 46%, third-line 40% vs. 43%), respectively. However, our rates of death are actually higher in both cohorts and across all lines of therapy. This finding is most likely attributed to differences in the definition of attrition in the studies. In the current study, we were able to define attrition more rigorously as patients who did not receive another line of treatment due to death or despite progressive disease. The US administrative study defined attrition as the ratio of patients who did not receive subsequent treatment due to death or lost to follow-up/no further treatment in follow-up [11]. This inherently overestimates attrition as it does not account for patients who are still alive but do not receive their next line of therapy due to ongoing response or disease stability. Using a MM-specific database with individual level patient data, we were able to capture those patients who remain on their prior therapy or are in remission off-treatment; such patients actually make up the bulk of those who have not yet received another line of treatment and, in turn, result in the lower attrition rate. This may be a more useful definition of attrition for informing stakeholders who need an accurate prediction of the number of patients expected to go on to further therapy.

A report from the disease-specific Australian Myeloma Registry on attrition in 571 MM patients was recently presented, and similarly defined attrition as patients not receiving further treatment due to death or despite progressive disease [13]. This study also showed lower attrition rates than the US report: 20% after line 1 with 37% having further treatment and 43% in remaining remission/on treatment, 19% after line 2 with 44% receiving subsequent treatment and 39% in remaining remission/on treatment, and 17% after line 3 with 42% having further treatment and 36% remaining remission/on treatment [13]. However, in contrast to our study, the Australian analysis did not separate ASCT and non-ASCT groups at each treatment line.

In addition to differing definitions of attrition, there may be additional factors contributing to the lower attrition rates seen in our study in Canada. Our patients are treated in generally standardized publicly reimbursed treatment pathways, with funded access to ASCT, bortezomib, carfilzomib, lenalidomide, pomalidomide, and daratumumab in various combinations. Within this type of system there may be lower actual and perceived “out of pocket” financial burden of treatment directly to patients, which could lead to more patients remaining on continuous therapy and/or being able to receive therapy at relapse. In addition, a high proportion of patients maintain treatment and follow-up at the same center throughout their illness.

Our results, as well as the US [11] and Australian [13] studies show higher rates of attrition in older patients not undergoing ASCT, which is expected given the increasing comorbidity burden and risk of frailty that can be seen with ageing. Multivariable analyses in both our study and the US analysis [11] confirmed older age as a predictor of attrition.

A more recent year of diagnosis was also associated with a reduced risk of attrition in our study, which may be a result of better supportive care measures or access to the more effective novel therapies which have been publicly reimbursed in Canada over this period. The Australian analysis observed a reduced risk of attrition with triplet and quadruplet regimens compared to PI-dexamethasone combinations, as well as reduced attrition risk associated with the use of maintenance lenalidomide [13]. In our study of over 5000 patients, we did not analyze the impact of individual treatment regimens on attrition rates.

Our study has several strengths including the large study population and disease-specific database, allowing for the capture of patient level data on rates of ongoing treatment and remission. Patients were treated in a publicly reimbursed health care system with similar standardized provincial treatment algorithms across the country that may minimize treatment-related differences potentially impacting attrition rates.

Our study also has some limitations. We were unable to capture certain patient characteristics that could be contribute to the risk for attrition, such as comorbidities, and we were unable to analyze the association of each treatment regimen with attrition. In addition, our follow-up time in the 2016–2020 cohort was not long enough (particularly for first line patients) to account for the impact of the most novel treatments on attrition rates. Our study, similar to other studies of attrition in MM [11], may underestimate the overall rate of attrition in patients with MM since we required patients to receive at least one line of treatment. As a result, we did not capture patients experiencing early mortality or opting for no treatment, therefore introducing immortal time bias. Lastly, attrition as defined by us and others [11] is agnostic to time on therapy- it is a rate of patients who do not receive a subsequent line of treatment. Therefore, a patient with aggressive disease who undergoes attrition after 4 lines of therapy may have the same OS as a patient with more indolent disease who has attrition after 1 line. Recognizing that patients with certain patient or disease characteristics may be at higher risk for experiencing attrition, we did perform a multivariable analysis to evaluate factors that could predict higher risk of attrition.

Nevertheless, this is the largest study of attrition rates in patients with MM using a disease-specific database with longitudinal individual patient level data. Our results support a revised definition of attrition which accounts for the fact that most patients who do not receive subsequent therapy are either continuing their current therapy and/or are in remission off-treatment—and therefore may be candidates for future regimens—rather than being lost to attrition. A more standardized approach to reporting such data is needed to better contextualize future research efforts in this area. The results of such analyzes may impact future policy discussions and decisions regarding the implementation of novel treatment strategies, particularly in universal health care systems.

### Supplementary information

Attrition by ASCT status and time cohort

## Data Availability

The datasets generated during and/or analyzed during the current study are not publicly available due to privacy laws but access is available through the corresponding author on reasonable request. For original data please contact amccurdy@toh.ca.
