# Correction: Redefining attrition in multiple myeloma (MM): a Canadian Myeloma Research Group (CMRG) analysis

**DOI:** 10.1038/s41408-023-00888-6

**Published:** 2023-08-28

**Authors:** Arleigh McCurdy, Hira Mian, Richard LeBlanc, Victor H. Jimenez-Zepeda, Jiandong Su, Esther Masih-Khan, Alissa Visram, Martha Louzada, Kevin Song, Darrell White, Michael Sebag, Julie Stakiw, Anthony Reiman, Muhammad Aslam, Debra Bergstrom, Rami Kotb, Rayan Kaedbey, Engin Gul, Donna Reece, Christopher P. Venner

**Affiliations:** 1 Ottawa Hospital Research Institute, The Ottawa Hospital, Ottawa, ON Canada; 2 Juravinski Cancer Center, Hamilton, ON Canada; 3 Maisonneuve-Rosemont Hospital Research Centre, University of Montreal, Montreal, QC Canada; 4 Arnie Charbonneau Cancer Institute, University of Calgary, Calgary, AB Canada; 5 Canadian Myeloma Research Group, Vaughn, ON Canada; 6 Princess Margaret Cancer Centre, Toronto, ON Canada; 7 Department of Medicine, London Regional Cancer Center, London, ON Canada; 8 BC Cancer Agency, Vancouver General Hospital, Vancouver, BC Canada; 9 Queen Elizabeth II Health Sciences Centre, Dalhousie University, Halifax, NS Canada; 10 Department of Medicine, McGill University, Montreal, QC Canada; 11 Saskatoon Cancer Centre, University of Saskatchewan, Saskatoon, SK Canada; 12 Department of Medicine, Saint John Regional Hospital, Saint John, NB Canada; 13 Department of Medical Oncology, Allan Blair Cancer Centre, Regina, SK Canada; 14 Division of Hematology, Memorial University of Newfoundland, St John’s, NL Canada; 15 Department of Medical Oncology & Hematology, Cancer Care Manitoba, Winnipeg, MB Canada; 16 Department of Medicine, Jewish General Hospital, Montreal, QC Canada; 17 Department of Medicine, Cross Cancer Institute, Edmonton, AB Canada

**Keywords:** Myeloma, Myeloma, Cancer epidemiology

Correction to: *Blood Cancer Journal* (2023) 13:111 10.1038/s41408-023-00883-x, published online 20 July 2023

In Table 2 of this article, the data in the columns headed “ASCT (*N* = 3111)” and “Non-ASCT (*N* = 2437)” were assigned in incorrect lines. Correct Table 2 is as follows:

**Table 2:** Multivariable model showing factors associated with attrition at each line of therapy by ASCT status.

LOT	Predictors	ASCT (*N* = 3111)	Non-ASCT (*N* = 2437)
		OR (95% CI)	OR (95% CI)
1	Sex:		
	Female Male	Ref **1.33 (1.00–1.78)**	Ref 1.20 (0.96–1.49)
	Age^a^:		
	<65 65–74	Ref **1.56 (1.16–2.09)**	Ref 0.95 (0.68–1.31)
	75–84	0 (0–Inf)	**1.54 (1.11–2.13)**
	>=85	0 (0–Inf)	**3.57 (2.38–5.35)**
	Cohort:		
	2010–2015 2016–2020	Ref **0.45 (0.33–0.63)**	Ref **0.70 (0.56–0.87)**
	Progression time (m):		
	≥24 12–24	Ref **1.59 (1.10–2.31)**	Ref **0.54 (0.38–0.79)**
	6–12	**2.81 (1.71–4.63)**	1.32 (0.94–1.86)
	<6	1.10 (0.51–2.36)	**3.45 (2.54–4.70)**
	Response:		
	<PR ≥PR	Ref 0.61 (0.35–1.08)	Ref **0.75 (0.56–1.00)**
2	Sex:		
	Female Male	Ref **1.44 (1.02–2.02)**	Ref **1.40 (1.08–1.81)**
	Age^a^:		
	<65 65–74	Ref **1.44 (1.01–2.07)**	Ref 1.01 (0.68–1.50)
	75–84	0 (0–Inf)	1.20 (0.80–1.79)
	>=85	0 (0–Inf)	**2.97 (1.72–5.15)**
	Cohort:		
	2010–2015 2016–2020	Ref 0.97 (0.68–1.40)	Ref **0.72 (0.55–0.94)**
	Progression time (m):		
	≥24 12–24	Ref 1.52 (0.83–2.76)	Ref 1.20 (0.81–1.77)
	6–12	**2.00 (1.17–3.43)**	1.34 (0.89–2.01)
	<6	**4.63 (3.07–7.00)**	**2.48 (1.78–3.46)**
	Response:		
	<PR ≥PR	Ref 1.40 (0.91–2.17)	Ref 0.76 (0.55–1.04)
	Gender: Female	Ref	Ref
	Male	1.11 (0.78–1.58)	1.13 (0.81–1.58)
3	Age^a^:		
	<65 65–74	Ref **1.78 (1.20–2.62)**	Ref 0.98 (0.60–1.62)
	75–84	0 (0–Inf)	1.04 (0.62–1.73)
	>=85	0 (0–Inf)	2.32 (0.92–6.01)
	Cohort:		
	2010–2015 2016–2020	Ref 1.13 (0.74–1.72)	Ref 0.92 (0.64–1.31)
	Progression time (m):		
	≥24 12–24	Ref 1.08 (0.51–2.27)	Ref **3.12 (1.74–5.59)**
	6–12	1.62 (0.92–2.84)	**2.44 (1.41–4.23)**
	<6	**2.80 (1.73–4.51)**	**3.94 (2.48–6.27)**
	Response:		
	<PR ≥PR	Ref 0.90 (0.90–1.37)	Ref **0.57 (0.38–0.86)**

Values bolded *p* < 0.05.

^a^Age at therapy initiation.

The original article has been corrected.

