# Supplementary material for: Redefining attrition in multiple myeloma (MM): a Canadian Myeloma Research Group (CMRG) analysis
Source: Blood Cancer J. 2023 Jul 20;13(1):111. doi: 10.1038/s41408-023-00883-x (PMC10359388; doi:10.1038/s41408-023-00883-x)
Supplement: Supplementary file 1 — Attrition by ASCT status and time cohort [file 41408_2023_883_MOESM1_ESM.pdf]

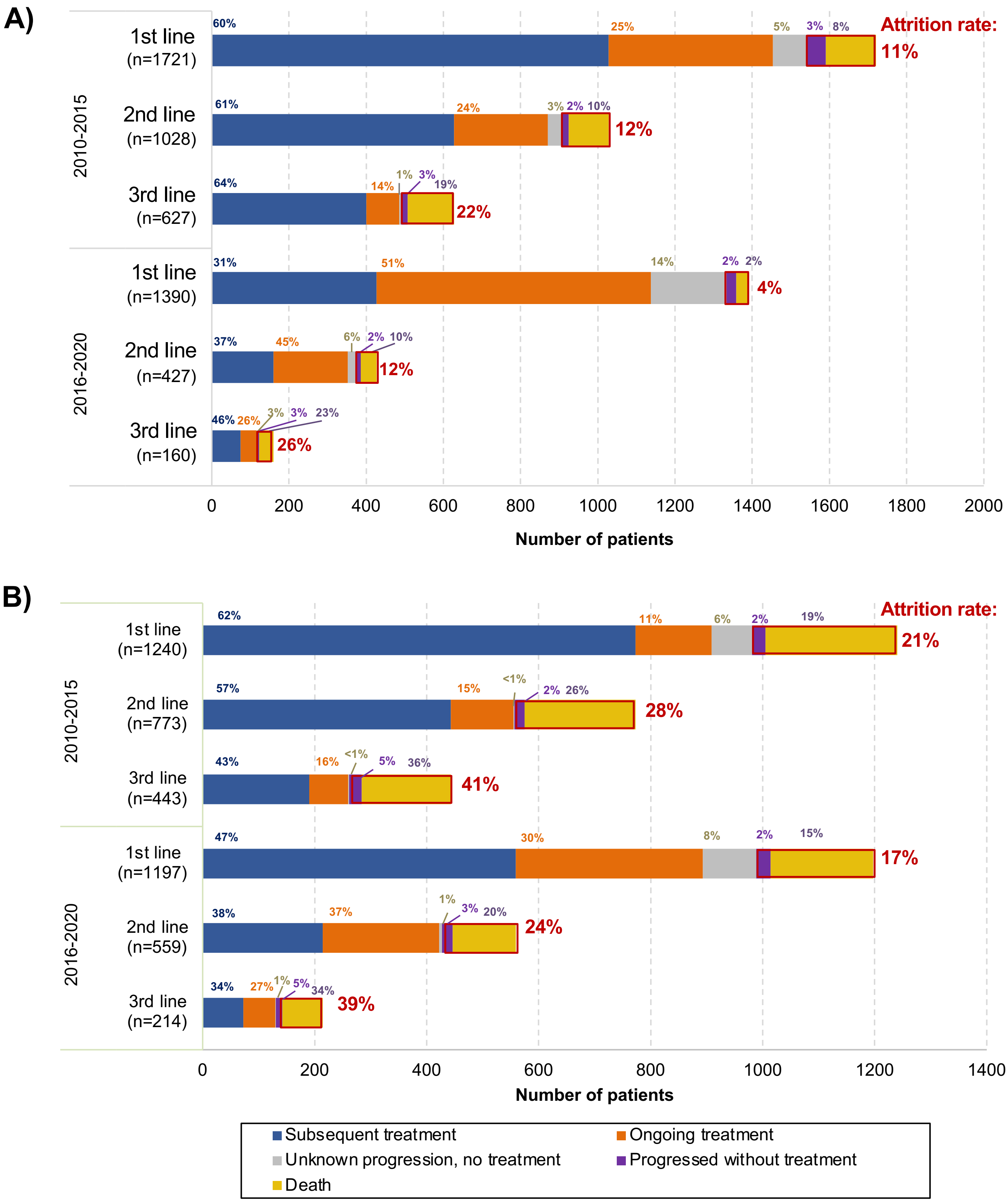

Figure 1S. Disposition of A) transplanted and B) non-transplanted patients with each subsequent line of treatment. Patient cohorts are stratified by date of diagnosis (2010-2015 and 2016-2020). Attrition with each line of therapy is shown in red, and the percentage of attrition if the percent of patients treated within each line of therapy that either died or progressed without receiving subsequent treatment.

Supplemental Table 1: Multivariable Model showing factors associated with attrition at each line of therapy by time cohort

LOT	Predictors	t_1= 2010-201 5 (n=2961)		t_2=2016-2020 (n=2587)	
		ASCT	Non-ASCT	ASCT	Non-ASCT
		OR(95% CI) *p* value	OR(95% CI) *p* value	OR(95% CI) *p* value	OR(95% CI) *p* value
1	Sex: Female	Ref	Ref	Ref	Ref
	Male	**1.48 (1.05-2.10)**	1.17 (0.87-1.59)	1.06 (0.61-1.85)	1.24 (0.90-1.72)
	Age*: < 65	Ref	Ref	Ref	Ref
	65-74	1.36 (0.95-1.96)	10.72 (0.47-1.08)	**2.02 (1.17-3.49)**	1.45 (0.83-2.52)
	75-84	0 (0-Inf)	1.24 (0.81-1.89)	0 (0-Inf)	**2.11 (1.22-3.65)**
	>=85	0 (0-Inf)	**3.12 (1.75-5.58)**	0 (0-Inf)	**4.59 (2.47-8.52)**
	Progression time (m): ≥24	Ref	Ref	Ref	Ref
	12-24	1.33 (0.88-2.02)	**0.47 (0.29-0.76)**	**2.83 (1.27-3.49)**	0.68 (0.38-1.22)
	6-12	**2.17 (1.20-3.95)**	1.07 (0.66-1.73)	**5.52 (2.17-14.02)**	**1.76 (1.08-2.88)**
	< 6	0.39 (0.12-1.31)	**2.97 (1.91-4.62)**	**5.00 (1.82-13.75)**	**4.33 (2.79-6.73)**
	Response: < PR	Ref	Ref	Ref	Ref
	≥PR	**0.42 (0.22-0.80)**	0.70 (0.47-1.04)	1.46 (0.40-5.32)	0.81 (0.52-1.26)
		1.85 (0.86-3.99)	0.88 (0.48-1.61)	1.17 (0.11-12.84)	1.40 (0.79-2.48)
2	Sex: Female	Ref	Ref	Ref	Ref
	Male	1.48 (0.98-2.24)	**1.40 (1.10-1.94)**	1.28 (0.68-2.39)	1.40 (0.91-2.14)
	Age*: < 65	Ref	Ref	Ref	Ref
	65-74	1.52 (0.98-2.35)	1.05 (0.63-1.73)	1.24 (0.65-2.36)	1.00 (0.52-1.92)
	75-84	0 (0-Inf)	1.31 (0.78-2.19)	0 (0-Inf)	1.05 (0.55-2.01)
	>=85	0 (0-Inf)	**3.75 (1.74-8.11)**	0 (0-Inf)	**2.45 (1.07-5.62)**
	Progression time (m): ≥24	Ref	Ref	Ref	Ref
	12-24	1.47 (0.75-2.89)	0.92 (0.58-1.47)	2.08 (0.56-7.78)	1.96 (0.98-3.94)
	6-12	**2.13 (1.15-3.95)**	1.01 (0.62-1.67)	1.59 (0.51-4.98)	**2.27 (1.11-4.66)**
	< 6 m	**4.58 (2.79-7.51)**	1.61 (1.03-2.52)	**5.20 (2.44-11.07)**	**4.29 (2.56-7.19)**
	Response: <PR	Ref	Ref	Ref	Ref
	≥PR	1.16 (0.71-1.92)	0.76 (0.51-1.15)	2.45 (1.01-5.96)	0.64 (0.38-1.08)
	Unknown	1.88 (0.97-3.61)	1.18 (0.65-2.13)	2.00 (0.65-6.13)	0.94 (0.48-1.82)
3	Sex: Female	Ref	Ref	Ref	Ref
	Male	1.20 (0.80-1.80)	0.99 (0.66-1.48)	0.93 (0.42-2.05)	1.66 (0.89-3.09)
	Age*: < 65	Ref	Ref	Ref	Ref
	65-74	**2.26 (1.45-3.53)**	0.91 (0.51-1.63)	1.01 (0.43-2.39)	1.54 (0.57-4.19)
	75-84	0 (0-Inf)	1.23 (0.66-2.29)	0 (0-Inf)	0.94 (0.35-2.54)
	>=85	0 (0-Inf)	**4.07 (1.07-15.41)**	0 (0-Inf)	1.69 (0.37-7.85)
	Progression time (m): ≥24	Ref	Ref	Ref	Ref
	12-24	0.88 (0.40-1.92)	**2.83 (1.41-5.68)**	3.95 (0.31-50.10)	**3.17 (1.04-9.711)**
	6-12	0.93 (0.49-1.79)	**2.22 (1.18-4.19)**	**12.74 (3.00-54.19)**	2.32 (0.71-7.56)
	< 6	**2.43 (1.42-4.15)**	**3.21 (1.82-5.67)**	**4.34 (1.27-14.86)**	**5.55 (2.41-12.78)**
	ResponseL3: <PR	Ref	Ref	Ref	Ref
	≥PR	0.92 (0.57-1.48)	**0.56 (0.34-0.92)**	0.58 (0.21-1.59)	0.53 (0.24-1.18)
	Unknown	1.11 (0.58-2.12)	1.67 (0.83-3.35)	0.82 (0.17-4.03)	0.91 (0.33-2.48)

Values bolded p<0.05
***age at therapy initiation**
